# Artificial light at night extends pollen season and elevates allergen exposure

**DOI:** 10.1093/pnasnexus/pgaf405

**Published:** 2026-01-20

**Authors:** Brandt Geist, Lin Meng, Daniel S W Katz, Huidong Li, Franz Hölker, Qian Xiao

**Affiliations:** Department of Earth and Environmental Sciences, Vanderbilt University, Nashville, TN 37235, USA; Department of Earth and Environmental Sciences, Vanderbilt University, Nashville, TN 37235, USA; School of Integrative Plant Science, Cornell University, Ithaca, NY 14853, USA; Department of Earth and Environmental Sciences, Vanderbilt University, Nashville, TN 37235, USA; Leibniz Institute of Freshwater Ecology and Inland Fisheries (IGB), Müggelseedamm 310, Berlin 12587, Germany; Institute of Biology, Freie Universität Berlin, Königin-Luise-Straße 1-3, Berlin 14195, Germany; Department of Epidemiology, Human Genetics and Environmental Sciences, School of Public Health, The University of Texas Health Science Center at Houston, Houston, TX 77030, USA; Center of Spatial-Temporal Modeling for Applications in Population Sciences, School of Public Health, The University of Texas Health Science Center at Houston, Houston, TX 77030, USA

**Keywords:** pollen, light pollution, public health, urban, phenology

## Abstract

Artificial light at night (ALAN), a growing environmental stressor in urban ecosystems, disrupts natural light–dark cycles and alters plant phenological events such as leaf-out and flowering. However, the extent to which ALAN influences airborne pollen season timing and exacerbates allergy-related health risks remains largely understudied. This study investigates how ALAN influences the timing and duration of the airborne pollen season across the Northeastern United States from 2012 to 2023 and the consequences of allergenic pollen exposure. Using daily pollen concentrations from the National Allergy Bureau, ALAN data from the Visible Infrared Imaging Radiometer Suite product, and gridded Daymet climate data, we derived three key pollen season metrics: start of season, end of season, and season length, and examined their relationship with environmental conditions. We found that higher ALAN exposure was significantly associated with an earlier start of pollen season, a later end of season, and a longer pollen season length, after controlling for temperature and precipitation. ALAN’s impact on the end of the season is larger than on the start of the season. ALAN sites experienced more days and higher severity for allergenic pollen exposure, relative to sites with minimal or no ALAN exposure. These results underscore the potential of ALAN to exacerbate allergy-related disease burdens, calling for its integration into urban environmental public health and planning strategies.

Significance Statement Artificial light at night (ALAN) is a rapidly expanding form of environmental stressor, yet its effects on pollen dynamics and allergy-related health risks remain largely unexamined. Using long-term airborne pollen data and satellite-derived ALAN observations, this study shows that ALAN is associated with shifting the timing and lengthening the duration of the pollen season across the Northeastern United States. These shifts lead to more days and higher severity of allergenic pollen exposure, suggesting that ALAN is an overlooked driver of allergy risk. The findings highlight the need to incorporate nighttime lighting into public health planning, particularly in urban areas where ALAN exposure and allergy prevalence are the highest.

## Introduction

Seasonal allergies and asthma have been pressing public health concerns in the United States, affecting tens of millions of individuals each year (1, 2). Current estimates indicate that between 10 and 25% of the population experience symptoms associated with allergic rhinitis or allergic asthma, with some agencies placing the broader burden of allergy-related health conditions at nearly one-third of the US adult population (3–5). Most aeroallergen exposure is driven by anemophilous (wind-pollinated) trees and grasses, which release large quantities of small, easily dispersed pollen grains (6, 7). Climate change, particularly rising temperatures, has been identified as a key driver of lengthening pollen seasons and intensifying pollen loads across North America (8, 9). Precipitation's effects on pollen are more variable, with short-term washout reducing airborne pollen but longer-term moisture availability potentially increasing pollen concentration for certain taxa (10). In cities, unique environmental conditions, including the urban heat island effect, elevated levels of air pollution, and increased exposure to artificial light at night (ALAN) may further affect pollen dynamics by directly influencing plant circadian rhythms as well as via interactions between plants and other species (11–13). Among these, ALAN remains an underexplored but potentially impactful factor that may shift pollen phenology and allergen exposure (14–17). In recent decades, artificial illumination has expanded rapidly across the globe, altering the natural diurnal light regime that has shaped plant evolution for millions of years (12, 18). Today, >80% of the world's population lives under light-polluted skies, and the continued expansion and intensification of ALAN are expected to exert increasingly pronounced impacts on plant phenology (18–20). Given these trends, understanding how ALAN affects pollen phenology is therefore critical for addressing the growing burden of allergy-related diseases in urban populations (21).

Plants rely on natural daylength (i.e. photoperiod) to regulate phenological events, such as budburst, flowering, and pollen release (22–24). ALAN disrupts these light cues by extending the perceived length of day and delaying the onset of darkness, activating light-sensitive pathways that advance flowering and prolong reproductive activity, thereby altering phenological responses (12, 21). In urban areas where ALAN is pervasive, plants may flower earlier, extend their reproductive periods, and delay fall senescence (15, 21, 25). These shifts in phenology may in turn lead to earlier, prolonged, or more intense airborne pollen seasons, increasing the cumulative duration and concentration of allergenic pollen in the atmosphere. Consequently, populations in light-polluted areas may experience heightened and extended exposure to aeroallergens, exacerbating risks for individuals with allergic respiratory conditions such as allergic rhinitis and asthma (3). However, the direct impact of ALAN on airborne pollen remains largely unexplored. Moreover, it is unclear whether ALAN acts independently or synergistically with other environmental variables such as temperature and precipitation to shift pollen season timing or exacerbate allergy risk by extending exposure windows (26–28). Addressing these knowledge gaps is essential for understanding the multifactorial drivers of allergenic pollen exposure in urban ecosystems and for informing public health and urban planning interventions.

The Northeastern United States, which encompasses major metropolitan areas such as New York City, Boston, and Philadelphia, is an ideal setting for examining the environmental drivers of pollen phenology and allergen exposure. This region is characterized by high levels of ALAN exposure, well-defined seasonal climate patterns that regulate phenological cycles, and large human populations vulnerable to allergy-related illnesses (11, 19). Recent public health data indicates that the Northeast has a disproportionately high burden of pollen-related conditions, based on the elevated number of emergency department visits for asthma (29). In addition, the region's mix of urban, suburban, and peri-urban landscapes allows for comparative analyses across varying gradients of ALAN exposure. Together, these factors make the Northeastern United States a highly relevant region for investigating how ALAN influences pollen season dynamics and exacerbates allergy-related health risks.

The goal of this study is to investigate how exposure to ALAN affects pollen season timing and allergen exposure across the Northeastern United States for 2012–2023. We answer the following research questions: (i) How does ALAN impact the start, end, and length of the pollen season? (ii) Do ALAN, temperature, and precipitation exhibit interactive effects on pollen phenology? (iii) Does ALAN increase pollen allergen exposure? We hypothesized that higher ALAN exposure would lead to an earlier start and later end to the pollen season, thereby extending its duration. As a result, we hypothesized that ALAN would increase the number of days classified as symptomatically significant (see Materials and methods). To address these questions, we integrated three major datasets: historical airborne pollen counts from the National Allergy Bureau (NAB) at 12 monitoring sites in the Northeastern United States, satellite nighttime light data from Visible Infrared Imaging Radiometer Suite (VIIRS) Day/Night Band (DNB), and daily temperature and precipitation records from Daymet. Using multiple linear regression and mixed-effects modeling approaches, we tested how ALAN and climatic variables (temperature and precipitation) influence key pollen metrics, including start of season, end of season, and season length, and the consequent allergen exposure. This study offers a regional-scale assessment of disentangling the independent and combined effects of ALAN and climate on airborne pollen dynamics, with important implications for both ecological research and public health planning.

## Results

### ALAN significantly alters pollen season timing and duration

**Fig. 1.**
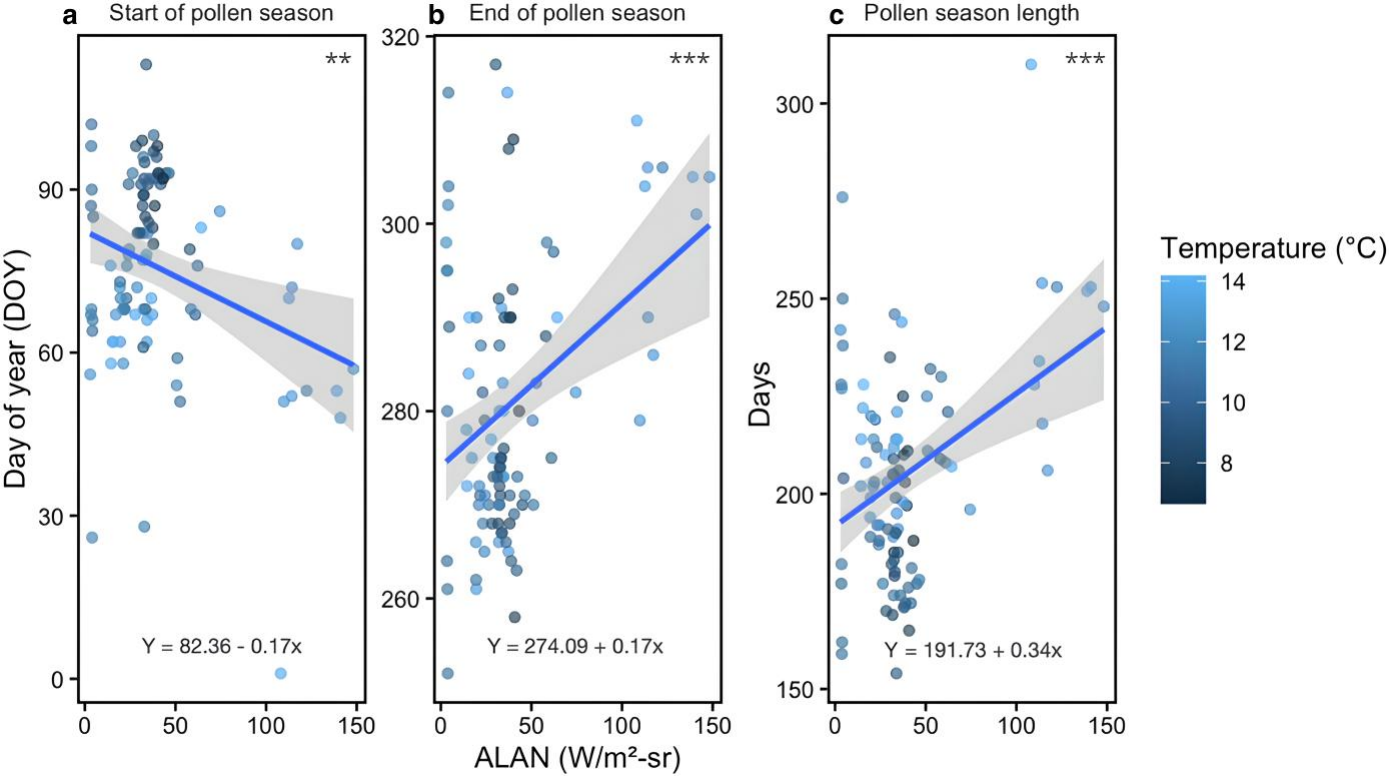
Relationship between ALAN and pollen season timing. Each point represents a pollen station-year combination for a) start of pollen season, b) end of pollen season, and c) pollen season length. Fitted linear regression line, 95% CI (shaded in gray), and fitted equations are shown in each panel. Statistical significance levels are shown in the upper right of each panel and denoted by asterisks: **P < 0.01, and ***P < 0.001.

We found the increase in ALAN is associated with both an earlier start of pollen release and a later end of pollen season, thereby substantially lengthening the pollen season (Fig. 1 and Table S1). The start of pollen season decreased significantly with increasing ALAN (Table S1, *β* = −0.166, *P* < 0.01), indicating an earlier onset under ALAN exposure. Most station-years with low ALAN (<25 nW/cm^2^/sr) had the start of seasons clustered around day-of-year (DOY) 70–100 (Fig. 1a). In contrast, those with high ALAN (>50 nW/cm^2^/sr) show the start of seasons ranging from DOY 50 to 80, indicating an advancement by around 20 days compared to low ALAN environments. Warmer temperatures further accentuated this early shift, with the earliest start of season appearing under the high ALAN and high temperature conditions. The end of season tends to occur significantly later under higher ALAN levels (Table S1, *β* = 0.174, *P* < 0.001). Sites with low ALAN showed end of seasons clustered between DOY 260 and 280, whereas sites in high ALAN environments were all beyond DOY 280 with values as late as DOY 310 (Fig. 1b, *P* < 0.001). Furthermore, the cooler sites exhibited an earlier end of pollen season. The combined effect of the earlier start of the season and the later end of the season carries over in the significant extension of the pollen season length with the increase in ALAN (Table S1, *β* = 0.340, *P* < 0.001). In low ALAN environments, season length generally ranged from 170 to 210 days (Fig. 1c, *P* < 0.001). In contrast, higher ALAN sites often exhibited season lengths well above 200 days, with some approaching or exceeding 250 days. We also tested two-way interactions between ALAN, temperature, and precipitation for each pollen metric (Table S2). None of the interaction terms were statistically significant, indicating that ALAN's associations with pollen season timing and duration were consistent across temperature and precipitation gradients. Thus, these findings support our hypothesis that ALAN advances the start and delays the end of pollen activity, extending the duration of the pollen season, especially under warm conditions.

**Fig. 2.**
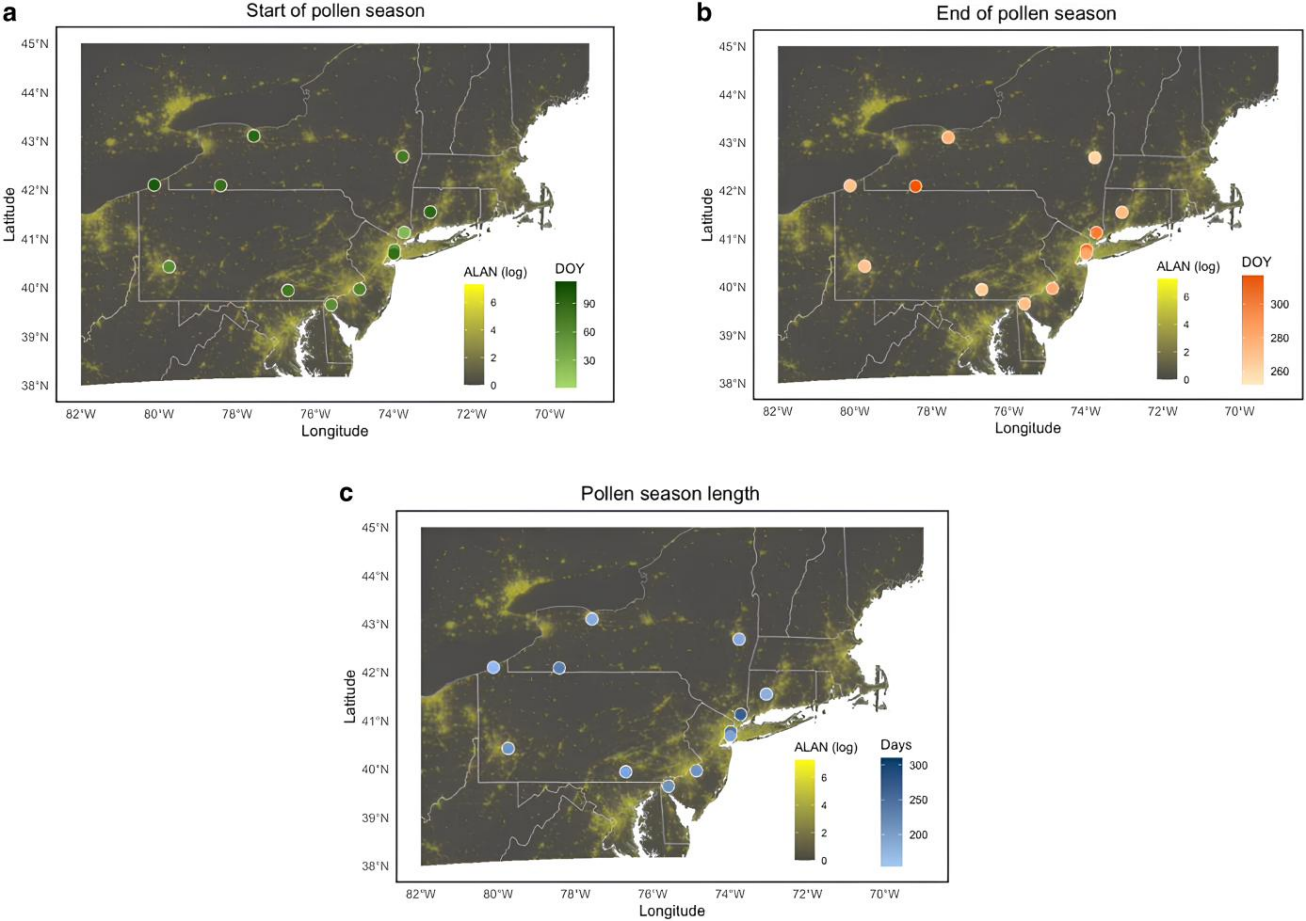
Spatial distribution of pollen phenology metrics and ALAN intensity across the Northeastern United States: a) start of pollen season, b) end of pollen season, and c) pollen season length. Points represent pollen monitoring sites colored by the average pollen metric across all study years. ALAN is displayed as a background continuous surface using a log-transformed color scale. White borders delineate US state boundaries.

The spatial pattern matches the regression trends seen in Fig. 1 and supports the hypothesis that ALAN exposure is linked to earlier start and later end of pollen season. Stations located in densely populated, highly illuminated areas, such as New York City, Philadelphia, and Pittsburgh, tend to have the earliest start of season, often before DOY 60 (Fig. 2a). In contrast, stations in less urbanized and illuminated areas, such as northwestern Pennsylvania, upstate New York, and rural Connecticut, exhibit later start of season dates, often around DOY 90. The pattern for the end of the season shows the opposite trend, with high ALAN areas like New York City experiencing delayed endings, some even extending beyond DOY 300 (Fig. 2b). Rural stations, particularly those in the inland regions, tend to have end of seasons around DOY 270 or earlier. For this reason, pollen activity is notably longer in urban centers, with season length often exceeding 240 days and in some cases nearing 300 days, whereas rural stations were typically concentrated between 200 and 240 days (Fig. 2c).

### Larger ALAN effects on the end of the pollen season than the start

The multiple regression results indicate that both ALAN and temperature are significant predictors of pollen season timing and duration, though their influence differs across specific pollen metrics (Table 1). ALAN was the only significant predictor of the end of the pollen season (*β* = 0.169, *P* < 0.001), suggesting that ALAN substantially delays the cessation of pollen activity, independent of climatic conditions. Although on a lower significance level, ALAN also advanced the start of the season (*β* = −0.099, *P* < 0.05) and lengthened the overall pollen season (*β* = 0.268, *P* < 0.001). Temperature strongly advanced the start of the pollen season (*β* = −5.38, *P* < 0.001) and also extended season length (*β* = 6.31, *P* < 0.001), consistent with previous understanding that warmer conditions promote earlier and longer periods of pollen release (8). In contrast, temperature showed no significant effect on the end of the season (*P* = 0.277). Total annual precipitation showed no statistically significant association with any pollen metric.

**Table 1.** Summary of linear regression models predicting start of pollen season, end of season, and season length.

Predictor	Start of season	End of season	Season length
ALAN	−0.10 (SE = 0.05, *t* = −2.11, *P* = 0.0378)	0.17 (SE = 0.05, *t* = 3.75, *P* = 0.0003)	0.27 (SE = 0.08, *t* = 3.52, *P* = 0.0007)
Temperature	−5.38 (SE = 0.89, *t* = −6.05, *P* < 0.0001)	0.93 (SE = 0.85, *t* = 1.09, *P* = 0.28)	6.31 (SE = 1.44, *t* = 4.39, *P* < 0.0001)
Precipitation	−0.17 (SE = 0.21, *t* = −0.79, *P* = 0.44)	−0.28 (SE = 0.20, *t* = −1.38, *P* = 0.17)	−0.11 (SE = 0.34, *t* = −0.33, *P* = 0.74)
Intercept	145.93 (SE = 11.32, *t* = 12.89, *P* < 0.0001)	275.20 (SE = 10.81, *t* = 25.47, *P* < 0.0001)	129.27 (SE = 18.28, *t* = 7.07, *P* < 0.0001)
Residual SE	14.36	13.71	23.19
*R* ^2^	0.37	0.17	0.31
Adjusted *R*^2^	0.35	0.14	0.29
*F*-statistic	18.18 (*P* < 0.0001)	6.10 (*P* = 0.0008)	13.60 (*P* < 0.0001)

Each column represents a separate model. Predictors include mean annual ALAN (nW/cm^2^/sr), mean annual temperature (°C), and total annual precipitation (mm).

To account for both environmental effects and heterogeneity across sites and interannual variability, we built linear mixed models with ALAN, temperature, and precipitation as fixed effects and station and year as random effects. The results were broadly consistent with those obtained from multiple linear regression models, indicating the robustness of the observed associations. Specifically, in the mixed model predicting the end of the season, ALAN was the only significant predictor (*β* = 0.186, *P* < 0.05, Table 2), an effect not observed for temperature (*P* = 0.198). ALAN also approached significance in the season length model (*β* = 0.250, *P* = 0.087). These suggest a consistent trend toward delaying the end of the season and lengthening the pollen season. In contrast, ALAN did not significantly influence the start of the season (*β* = −0.081, *P* = 0.299). Temperature remained a significant predictor of earlier pollen season onset (*β* = −5.242, *P* < 0.01) and longer season length (*β* = 6.751, *P* < 0.01). Precipitation was not significant for the start of the season or season length but had a significant negative association with the end of the season (*β* = −0.418, *P* < 0.05). Variance estimates showed that station-level effects explained a large portion of the variability (start of season: 36.69 days; end of season: 61.76 days; season length: 156.41 days), indicating substantial site-level differences in pollen dynamics. Year-to-year variability was comparatively much smaller (start of season: 8.30 days; end of season: 0.37 days; season length: 25.35 days). Overall, these models highlight ALAN as a distinct and robust predictor of pollen phenological change, especially for the end of pollen season.

**Table 2.** Summary statistics for each pollen timing metric (start of season, end of season, and season length) based on linear mixed-effects models.

	Variables	Start of season	End of season	Season length
Fixed effects	ALAN	−0.08 (0.07)	0.19 (0.08)	0.25 (0.13)
	Temperature	−5.24 (1.26)	1.64 (1.22)	6.75 (2.21)
	Precipitation	−0.18 (0.23)	−0.42 (0.19)	−0.21 (0.36)
	Intercept	144.84 (16.04)	271.09 (16.04)	127.18 (28.14)
Random effects	Station	36.69 (6.06)	61.76 (7.86)	156.41 (12.51)
	Year	8.30 (2.88)	0.37 (0.61)	25.35 (5.03)
	Residual	176.02 (13.27)	135.65 (11.65)	395.07 (19.88)
*P*-values	ALAN	0.299	0.039	0.087
	Temperature	0.002	0.198	0.007
	Precipitation	0.455	0.041	0.568

The table includes the variance estimates for random effects (station, year, and residual) and fixed effects estimates for mean annual ALAN (nW/cm^2^/sr), mean annual temperature (°C), and total annual precipitation (mm). The values in parentheses represent standard errors for fixed effects and one SD for random effects.

### ALAN increases allergy exposure severity

**Fig. 3.**
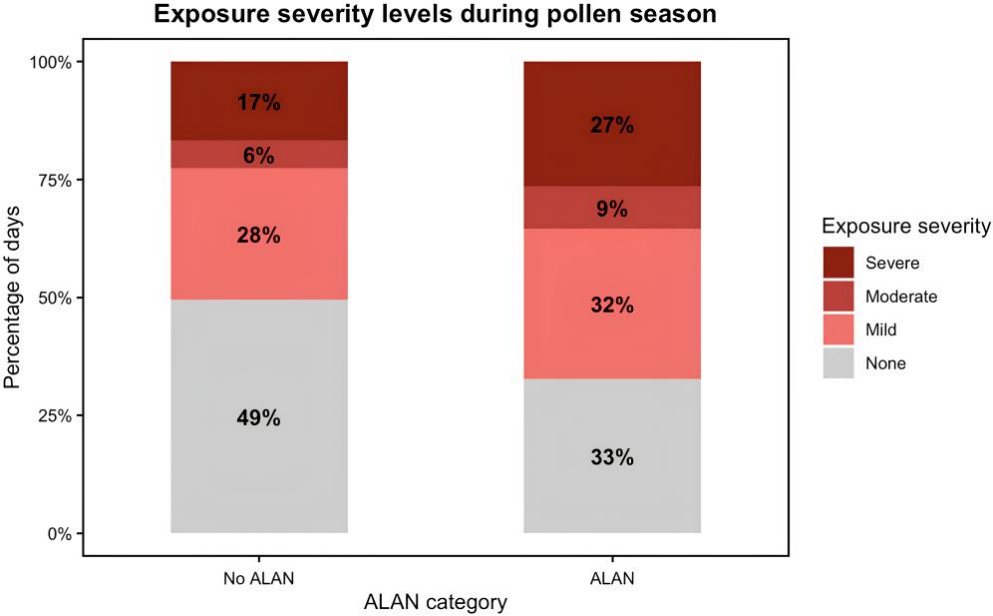
Proportion of days in the pollen season under four exposure severity levels for “No ALAN” and “ALAN” conditions. Pollen station-year combinations were classified as either “No ALAN” (<10 nW/cm^2^/sr) or “ALAN” (≥10 nW/cm^2^/sr) based on their ALAN intensity. Allergy severity was assigned according to total daily pollen concentrations: “None” (<10 grains/m^3^), “Mild” (10–49 grains/m^3^), “Moderate” (50–89 grains/m^3^), and “Severe” (≥90 grains/m^3^).

To assess potential health implications of ALAN, we categorized each day of the pollen season into one of four allergy exposure severity levels based on the total daily pollen concentrations—none (<10 grains/m^3^), mild (10–49 grains/m^3^), moderate (50–89 grains/m^3^), or severe (≥90 grains/m^3^)—and established symptom-triggering thresholds (30–34). This classification allowed us to evaluate how ALAN exposure affects not just the timing of pollen release but also the intensity of allergen exposure. In ALAN-exposed environments, a larger percentage of pollen season days fell into higher exposure severity categories compared to areas without ALAN (Fig. 3). Notably, 27% of pollen season days were classified as causing “severe” exposure under ALAN conditions, compared to just 17% in areas with little to no ALAN. Days with “moderate” exposure accounted for 9% of the season in ALAN-exposed sites versus 6% at non-ALAN sites. Days with “mild” exposure were also more frequent in ALAN areas at 32% compared to 28% in areas without ALAN. In contrast, “none” days (those below symptom-triggering thresholds) comprised only 33% of the season under ALAN, compared to 50% where ALAN was minimal or absent. This shift in the distribution of exposure severity levels indicates that ALAN influences not only the timing and duration of the pollen season but also its daily intensity, with implications for public health planning and allergy management.

## Discussion

Our findings reveal that ALAN may be a significant driver of environmental change, influencing the timing and intensity of seasonal pollen exposure through changes in pollen phenology. By extending the pollen season and elevating pollen concentrations, ALAN may modulate plant biological rhythms and amplify allergen exposure in urban environments, an underrecognized consequence of light pollution with implications for both ecosystem functioning and public health.

Our finding aligns with previous studies showing that ALAN can mimic longer photoperiods and thus accelerate spring phenology, delay leaf senescence, and extend flowering (15, 21, 25). Light-sensitive processes such as flowering and reproductive development in many plant species are regulated by photoreceptors that respond to daylength and light intensity (23, 24). ALAN, by artificially extending perceived daylength, likely disrupts these natural photoperiod cues, thereby inducing earlier flowering and, consequently, earlier pollen release. The extension of the pollen season may be due to the continued physiological stimulation of plants under prolonged artificial lighting, potentially delaying senescence or suppressing photoperiod-induced dormancy signals (35). By increasing the number of flowering days and potentially enhancing total floral and reproductive output, ALAN exposure may also increase cumulative pollen production, leading to higher airborne pollen concentrations over the season. Notably, ALAN remained a significant predictor of the end of the pollen season even after accounting for spatial and temporal variation in the linear mixed-effects model. This robustness across different locations and years suggests that the influence of ALAN on extending pollen activity is not limited to specific microclimates or urban configurations. Rather, it points to a more generalizable effect of ALAN on delaying the end of allergenic pollen exposure. The significant effects of temperature on both start of season and season length align with the established relationship of warmer temperatures advancing phenological events and extending the growing season (8, 36–38).

The increased allergen exposure in ALAN-affected areas suggests that phenological shifts induced by ALAN could be contributing to increased health risk and exposure burden for allergy sufferers (9, 39, 40). In our analysis, the thresholds for exposure severity are applied to total airborne pollen as an indicator of “high-exposure” days, while recognizing allergenicity varies among taxa. Notably, ALAN has been linked to reduced sleep quality, while allergenic conditions can also impair sleep, potentially worsening the health circumstances in affected groups (41–43). Thus, our results are especially important for urban planning related to public health, where both ALAN exposure and population density are high (44, 45). These results build on existing evidence that ALAN can have wide-ranging ecological impacts (14, 46, 47). While much of the literature has focused on animals (e.g. insects, birds, and bats) (48), this study demonstrates that plant seasonal dynamics—particularly those with direct consequences for human health—are also susceptible to ALAN. Furthermore, this study provides a new insight into the relationship between ALAN and plant reproductive phenology, suggesting that ALAN may act as another relevant environmental stressor alongside temperature and precipitation. Such stressors shape biological timing, which urban planners and policymakers should consider when formulating strategies to address the unintended ecological and health consequences of excessive nighttime lighting.

Our findings hold direct implications for urban tree planning, given the interaction between tree species' allergenic pollen production and their sensitivity to photoperiod cues. Many allergenic tree species commonly planted in cities, such as *Quercus* (oak), *Morus* (mulberries), *Platanus* (sycamore), and *Ulmus* (elm), are major contributors to airborne pollen loads and are known to be responsive to photoperiod cues (49). Photoperiod-sensitive species may be particularly vulnerable to phenological disruption under ALAN (25). In contrast, some early-successional species, which often exhibit weaker photoperiodic control, may be less affected by ALAN exposure and may offer more stable flowering phenology under urban light conditions (15). These differences highlight a potential value in considering not only allergenicity but also photoperiod sensitivity when selecting tree species for planting in light-polluted environments. Strategic selection of lower-allergen, less photoperiod-sensitive species, especially near residential areas, schools, and healthcare facilities, could help reduce allergen exposure (50).

Light pollution mitigation strategies, such as shielding fixtures, reducing light levels, using motion-sensor lighting, and minimizing blue-spectrum light emissions, could help to reduce the impacts on urban vegetation and reduce the health burden of allergenic sources (51–53). Lastly, allergy forecasting systems and public health advisories may need to integrate ALAN exposure metrics into their models to better predict pollen season onset and severity. Incorporating ALAN data into these systems would allow for more accurate identification of high-risk periods and areas, especially in densely populated urban areas where both light pollution and allergy prevalence are elevated (21). This could enhance early warning capabilities, improve individual symptom management, and inform city-level public health responses during peak allergy seasons (11).

Our findings were consistent across a 12-year period and multiple geographically diverse stations, underscoring the robustness of ALAN's association with pollen season timing. The use of both fixed and mixed-effects models further strengthen confidence in the results, particularly the persistent influence of ALAN on delaying the end of the pollen season. While generalizability may be constrained by the limited number of monitoring stations and reliance on interpolated pollen data, the spatial and temporal consistency observed highlights a strong and repeatable signal. Future research should explore more rigorous causal designs (e.g. quasi-experimental approaches or station-stratified reshuffling tests), examine whether similar patterns emerge in other regions with different climates and species compositions, and explore how other environmental factors like air pollution interacts with ALAN (54–56). In addition, future work could analyze ALAN, temperature, and precipitation at daily or monthly temporal resolutions to disentangle short-term lagged and cumulative effects and to identify critical windows when these drivers most strongly influence pollen season onset and cessation (20, 21). Controlled experiments would help clarify causal mechanisms, while linking ALAN exposure to health outcomes could enhance the policy relevance of this work (12, 52, 57). Future research should also account for potential biases in gridded climate datasets, as underestimation of urban temperatures in products like Daymet may cause ALAN to partially proxy urban heat (58, 59).

Taken together, our findings reveal that ALAN can significantly alter pollen phenology in ways that extend the duration of allergen exposure as well as allergy severity. These phenological shifts, while rooted in biological response mechanisms, carry far-reaching consequences for human health, particularly in densely populated urban areas where both light pollution and vulnerability to respiratory illnesses are high (60). By demonstrating that ALAN influences not only the timing but also the health relevance of plant seasonal dynamics, this work bridges ecological and epidemiological domains and highlights the urgency of integrating light pollution into environmental health assessments, allergy forecasting systems, and urban planning frameworks. The results emphasize that managing light at night is not solely an issue of energy efficiency or ecological conservation, but also of public health and urban resilience (26, 52, 53). As cities grow and artificial illumination continues to intensify, interdisciplinary strategies that align urban forestry, lighting design, and health policy will be essential for creating healthier, more sustainable, and ecologically informed urban environments (20, 46).

## Materials and methods

### Study area

This study focused on the Northeastern United States, a region encompassing densely populated metropolitan areas such as New York City, Boston, and Philadelphia, as well as surrounding suburban and rural zones. The region experiences a temperate climate with four distinct seasons, making it ideal for studying seasonal phenological shifts. Common allergenic tree species that dominate pollen production in this area include oaks (*Quercus* spp.), birches (*Betula* spp.), maples (*Acer* spp.), and elms (*Ulmus* spp.), with tree pollen typically peaking in early to mid-spring (30). Grass pollen peaks follow in late spring to early summer, while weed pollen, including ragweed (*Ambrosia artemisiifolia*), reaches its highest concentrations in late summer and early fall (30). The region also ranks among the most urbanized and light polluted in the country, with satellite-derived data showing elevated nighttime radiance in urban cores and along major transportation corridors (19). This region therefore provides a valuable gradient of ALAN exposure across a mix of urban, suburban, and rural environments, allowing for the investigation of its potential influence on pollen dynamics.

### Data description

#### ALAN data

ALAN data were obtained from NASA's Black Marble product (VIIRS VNP46A2), which provides daily global nighttime radiance measurements from the VIIRS DNB at a spatial resolution of 500 m (61). The data are radiometrically calibrated, BRDF corrected, cloud masked, and gap filled, offering consistent and high-quality observations in units of radiance (nW/cm^2^/sr) (62). We extracted ALAN data for each pollen station and calculated monthly average radiance for each year between 2012 and 2023. Although pollen collected at a station originates from surrounding areas and can at times include pollen from regional sources, local sources tend to drive airborne pollen dynamics in cities (63–65). We therefore utilized ALAN measurements directly from the location of the monitoring station to best represent the light environment influencing the observed concentrations. Monthly mean values were aggregated to compute seasonal and annual averages for each pollen monitoring station used in the models. Outliers in ALAN were identified using the *Z*-score method; values with *Z*-scores >2 were excluded from the analysis to remove extreme radiance.

#### Pollen data

The NAB dataset provides detailed measurements of pollen concentrations across the United States (66). The NAB operates the largest certified pollen monitoring network in North America, with 70 active stations reporting daily or near-daily pollen counts during the pollen season. The dataset spans back to 2003 and encompasses a broad range of allergenically important groups, including trees, grasses, weeds, and fungi (Table S3). Airborne pollen is monitored using volumetric spore traps and involves pulling a known volume of air through the device with the pollen adhering to a sampling surface. After collection, trained and certified pollen technicians analyze the samples under a microscope by manually identifying and counting pollen grains based on their unique morphological characteristics. Once the pollen grains are counted, the data are converted into pollen concentration values and expressed in grains per cubic meter of air (grains/m^3^). The full process follows standardized protocols set by the American Academy of Allergy, Asthma and Immunology to ensure accuracy and consistency across NAB stations (67). Twelve pollen stations were included in the analysis of this study (Table 3).

**Table 3.** List of pollen monitoring stations across the northeastern United States from the NAB.

Station name	City	State	Latitude	Longitude
The Louis Calder Center	Armonk	NY	41.1299	−73.7288
Certified Allergy Consultants	Albany	NY	42.6832	−73.7701
Long Island College Hospital	Brooklyn	NY	40.6904	−73.9970
Allergy and Asthma Associates of Northwestern PA	Erie	PA	42.1024	−80.1296
Division of Allergy, Asthma and Immunology, East Suburban Pediatrics	Monroeville	PA	40.4281	−79.7471
Allergy and Asthma Specialty Physicians, LLC	Mount Laurel	NJ	39.9693	−74.8716
Division of Air Quality, DNREC, State of Delaware	New Castle	DE	39.6488	−75.5981
Fordham College at Lincoln Center	New York	NY	40.7708	−73.9851
Fred H Lewis, MD FAAAAI	Olean	NY	42.0906	−78.4274
Allergy, Asthma & Immunology of Rochester	Rochester	NY	43.1017	−77.5817
Christopher Randolph, MD FAAAAI	Waterbury	CT	41.5496	−73.0659
Allergy and Asthma Consultants, Inc.	York	PA	39.9434	−76.7066

Each station is identified by name, city, and state, along with its latitude and longitude coordinates.

#### Climate data

Climate variables were obtained from the Daymet V4 dataset, which provides 1 km gridded daily surface weather data across North America (68). For this study, we used daily maximum temperature (*t*_max_), minimum temperature (*t*_min_), and precipitation (prcp) as predictors of pollen season timing. The Daymet data are derived from a network of ground-based meteorological stations and interpolated using terrain-sensitive algorithms to provide continuous spatial coverage. Although Daymet may underestimate temperatures in densely built urban areas due to limited urban stations, it remains one of the most reliable and widely validated sources of high-resolution climate data for ecological and environmental applications (58, 59). We extracted monthly mean *t*_max_ and *t*_min_ values, along with monthly total precipitation, for each pollen monitoring station from 2012 to 2023. These values were then aggregated to produce annual mean temperature and annual total precipitation for each station of each year. These annual climate metrics were used as covariates in statistical models to assess their independent and combined effects with ALAN on pollen season metrics.

### Pollen data preprocessing

To ensure consistency and reliability, we applied the following filters to the raw pollen data: (i) removed all observations belonging to the fungi group and the “unidentified pollen,” (ii) retained only stations with ≥5 years of data, (iii) excluded station-year combinations with <10 total measurements, and (iv) removed years lacking pollen counts both before April 15 and after September 15 to ensure adequate seasonal coverage. Since NAB stations are not monitored for the same number of years, our analyses are based on station-years, and a station contributes data only in years that meet these completeness criteria. To enable group-level analysis, we aggregated observations into three groups (i.e. trees, grasses, and weeds) using NAB's classification. A linear interpolation was conducted within each pollen season to estimate daily values for total pollen and for each of the three pollen groups. We then calculated the daily total pollen count with the interpolated data by summing all taxa for each day within the pollen season. This interpolation helped address irregular sampling frequencies and enabled continuous analysis across the growing season. To assess the validity of the interpolation, we aggregated both the daily interpolated and raw pollen data to the weekly level for direct comparison and found a strong alignment in seasonal patterns and magnitudes (Fig. S1). While excluding all station-years with gaps longer than a week could improve interpolation accuracy, it would substantially reduce the dataset size and spatial coverage. Our sensitivity analysis of station-years with the longest gaps (>7 days) showed that exclusion changed median start of season dates by 0 days, end of season dates by 0.5 days, and season length by 4 days. We therefore retained all station-years to preserve statistical power and representativeness. Pollen data prior to 2012 were excluded to align with the temporal availability of ALAN data. The resulting output is a dataset with 21,542 records across 12 pollen stations throughout the Northeastern United States covering a 12-year span (2012–2023).

### Deriving pollen metrics

In addition to using interpolated pollen data to calculate seasonal metrics such as total pollen, we applied a percentile-based thresholding method to determine the temporal bounds of the pollen season. Specifically, we identified the 30th percentile of daily total pollen concentrations for each station across all years as a threshold. Using a station-specific percentile rather than an absolute concentration helps avoid biases arising from differences in taxa, absolute abundance, and sampler efficiency, and the 30th percentile effectively filters out low-abundance winter values while capturing the onset of sustained seasonal pollen production. This approach accounts for spatial differences in pollen magnitude among stations while capturing the biologically and epidemiologically meaningful level above which pollen concentrations are likely to affect sensitive individuals. Similar percentile-based thresholds have been used in prior aerobiological studies, and previous sensitivity analyses indicate that results are robust to threshold choices between the 20th and 40th percentiles (8). For each station and year, days where total pollen exceeded this threshold are considered part of the pollen season. The first and last days in a given year with pollen above the threshold were designated as the start and end of the pollen season, respectively. Pollen season length was then computed as the number of days between these two dates. This approach standardizes pollen season metrics across stations with different baseline pollen levels and sampling frequencies, allowing for consistent comparisons of seasonal dynamics.

### Statistical analysis

Environmental metrics for ALAN, temperature, and precipitation data at the pollen stations were derived using Google Earth Engine. The final dataset included three main predictor variables (ALAN, temperature, and precipitation) and three response variables (start of pollen season, end of pollen season, and pollen season length) derived from the interpolated daily pollen data. To align the predictors with these responses, we summarized ALAN and climate at the same station-year resolution. Specifically, we computed annual mean nighttime radiance from monthly values, annual mean temperature from monthly mean *t*_max_ and *t*_min_, as well as annual total precipitation from monthly precipitation sums at each station. This annual aggregation reflects our focus on spatial and interannual variability in pollen phenology, which integrates the cumulative influence of light and climate over the full season rather than day-to-day fluctuations.

To evaluate the individual and combined effects of ALAN, temperature, and precipitation on pollen season timing, we fit separate multiple linear regression models for each of the three response variables: start of pollen season, end of pollen season, and pollen season length. The degrees of freedom for each model were 3 and 91, corresponding to three predictors and 91 station-year combinations as observations. The predictor variables included mean annual ALAN (nW/cm^2^/sr), mean annual temperature (°C), and total annual precipitation (mm), all of which were treated as continuous covariates. We used annual means (for ALAN and temperature) and annual sums (for precipitation) to capture full-season environmental conditions and provide a stable indicator of each site's typical light and climate regime. In addition to the main-effects models, we also tested all possible interactions between ALAN, temperature, and precipitation for each pollen metric to assess whether ALAN's effects varied across climate gradients (Table S2). Each model was fit using the base “lm()” function in R, with one pollen metric as the dependent variable and the three environmental predictors included simultaneously. To ensure that assumptions of linear regression were met, we assessed residual plots for normality and homoscedasticity and checked for multicollinearity among predictors using variance inflation factors (Table S4 and Fig. S2). We also confirmed there was only a weak positive correlation between ALAN and temperature with a Pearson correlation test (*R* = 0.22, *P* = 0.034). No transformations were applied, as visual and statistical diagnostics indicated that the assumptions were reasonably satisfied.

We first evaluated whether the relationship between ALAN and pollen metrics was nonlinear by comparing generalized additive models with a smooth term for ALAN (*k* = 5) and station random intercepts to models with a linear ALAN term. Similar fits and overlapping CIs (Table S5) indicated that a linear effect of ALAN was adequate. To test whether our ALAN estimates were driven by a few extreme observations, we calculated Cook's distance (threshold 4/*n*), refit models excluding flagged points, and also fit Huber-robust regressions. ALAN coefficients remained similar across these specifications (Table S6), suggesting that results are not sensitive to influential outliers. Finally, to distinguish contemporaneous from delayed ALAN effects, we compared models with current-year versus 1-year lagged ALAN using station fixed effects and year dummies, selecting between them using ΔAkaike Information Criterion (AIC). Lag tests showed similar support for the end of the season and season length (|ΔAIC| < 2), whereas contemporaneous ALAN provided a better fit for the start of the season (ΔAIC = 3.6; Table S7), consistent with a primarily same-year response of pollen onset to ALAN. Lastly, a station-level time-series analysis of ALAN revealed no significant long-term trend at most pollen stations, with only a few sites exhibiting notable increases or decreases, indicating that our results are not driven by systematic changes in ALAN over the study period (Fig. S3 and Table S8).

To account for repeated measurements and potential nonindependence across years and stations, we fit linear mixed-effects models using the “lme4” package in R. The models included fixed effects for mean annual ALAN, mean annual temperature, and total annual precipitation. Random intercepts for station and year were included to account for hierarchical structure in the data and unobserved variability at both spatial and temporal scales. This modeling approach enabled more accurate estimation of fixed effects while appropriately controlling for nested and repeated observations. Lastly, to ensure similar taxa contributed to pollen concentrations across sites, we compared taxonomic composition among stations using Bray–Curtis dissimilarity calculated from Hellinger-transformed pollen count data. The Hellinger transformation standardizes the data by eliminating magnitude differences between sites, reducing bias from dominant taxa, and enabling a more balanced assessment of community composition that accounts for both common and rare taxa (Table S9).

### Categorization of exposure levels under ALAN

To translate pollen exposure into a metric tied to human health, we derived daily pollen exposure severity levels based on daily total pollen concentrations. Each daily total pollen concentration value within the defined pollen season of every station-year combination was treated as a single record. Using established thresholds for daily total pollen concentrations, each record was categorized into one of four severity levels: “none” (<10 pollen grains/m^3^), “mild” (10–49), “moderate” (50–89), and “severe” (≥90) (30–34). To assess differences in allergy exposure by ALAN, we also classified each of these records into one of two categories—“ALAN” (>10 nW/cm^2^/sr) or “no ALAN” (<10 nW/cm^2^/sr)—based on the annual mean ALAN for each site. We then compiled all the records and grouped the data by ALAN category and exposure severity level (Fig. S4). For each ALAN group, we calculated both the total number of days that fell into each severity category and the relative percentage of days in that category. Relative frequency was computed by dividing the number of days in a given exposure severity class by the total number of records within that ALAN group. This approach provides a foundation for linking environmental conditions to potential allergen exposure and public health outcomes.

## Supplementary Material

pgaf405_Supplementary_Data

## Data Availability

All the data necessary to evaluate the results in this paper are present in the paper and/or Supplementary material. All the codes and derived data products to conduct the analyses are available on Open Science Framework at https://osf.io/hzrx9/?view_only=1f33ebc6b24c471d890ffdf558700dca.
