# Supplementary material for: Artificial light at night extends pollen season and elevates allergen exposure
Source: PNAS Nexus. 2026 Jan 20;5(1):pgaf405. doi: 10.1093/pnasnexus/pgaf405 (PMC12817216; doi:10.1093/pnasnexus/pgaf405)
Supplement: pgaf405_Supplementary_Data [file pgaf405_supplementary_data.pdf]

Supplementary Materials

Artificial light at night extends pollen season and elevates allergen exposure

Brandt Geist[1], Lin Meng[1*], Daniel S.W. Katz[2], Huidong Li[1], Franz Hölker[3,4], Qian Xiao[5,6]

[1] Department of Earth and Environmental Sciences, Vanderbilt University, Nashville, TN, 37235, USA
[2] School of Integrative Plant Science, Cornell University, Ithaca, NY, 14853, USA
[3] Leibniz Institute of Freshwater Ecology and Inland Fisheries (IGB), Müggelseedamm 310, 12587, Berlin, Germany
[4] Institute of Biology, Freie Universität Berlin, Königin-Luise-Straße 1-3, 14195, Berlin, Germany
[5] Department of Epidemiology, Human Genetics and Environmental Sciences, School of Public Health, The University of Texas Health Science Center at Houston, Houston, TX, 77030, USA
[6] Center of Spatial-Temporal Modeling for Applications in Population Sciences, School of Public Health, The University of Texas Health Science Center at Houston, Houston, TX, 77030, USA

[*] Corresponding Author: Lin Meng (lin.meng@vanderbilt.edu)

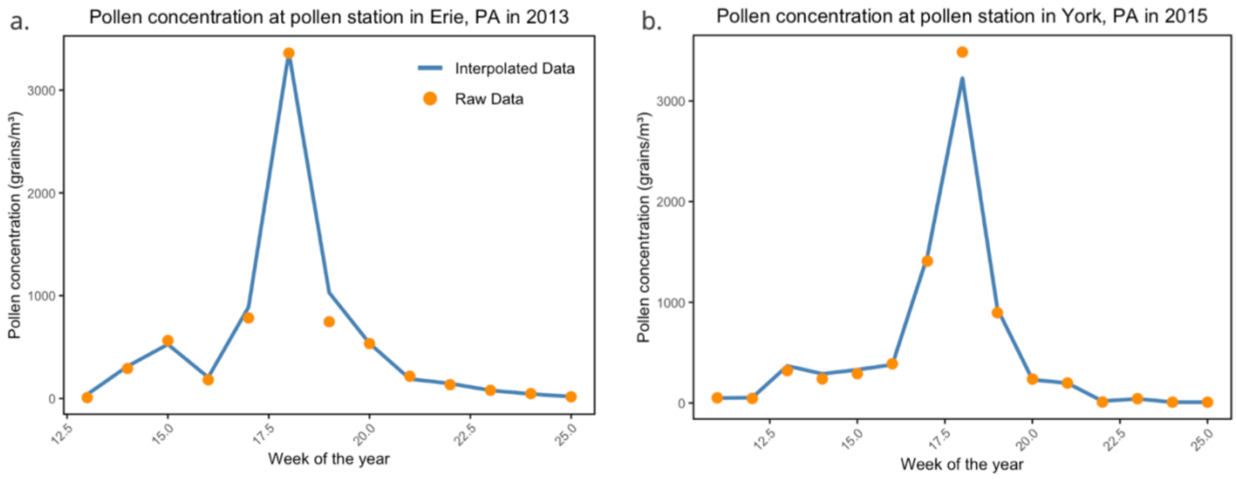

Fig. S1. Weekly average pollen concentrations (grains/m³) for two pollen stations: Erie, PA in 2013 (left) and York, PA in 2015 (right). Interpolation was performed to generate continuous daily pollen curves from discrete observations.

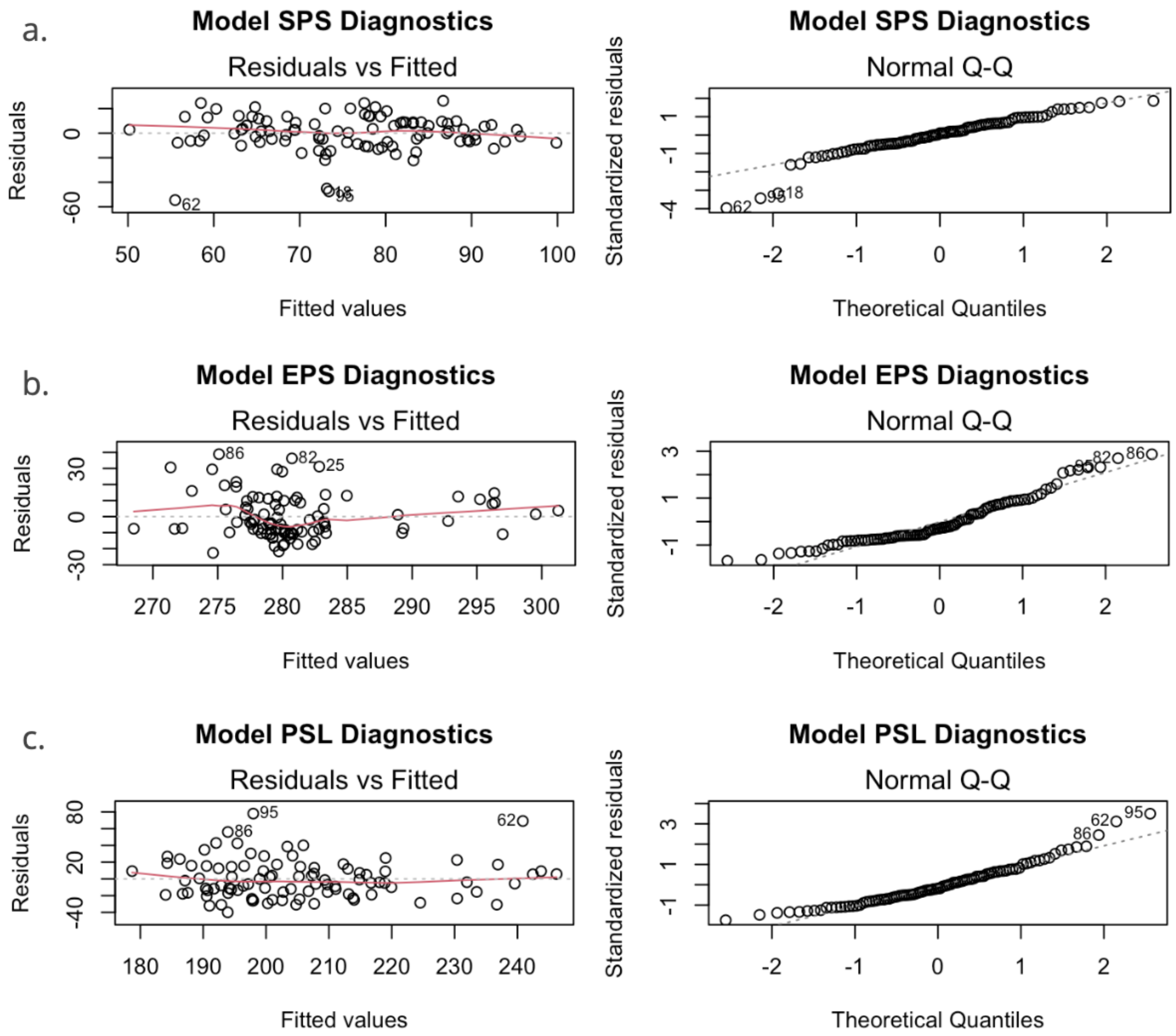

Fig. S2. Diagnostic plots for linear regression models. Residuals vs. Fitted plots (left) and Normal Q-Q plots (right) are shown for three models: (a) start of pollen season (SPS) model, (b) end of pollen season (EPS) model, and (c) pollen season length (PSL) model. These plots are used to assess the assumptions of linearity, homoscedasticity, and normality of residuals for each model.

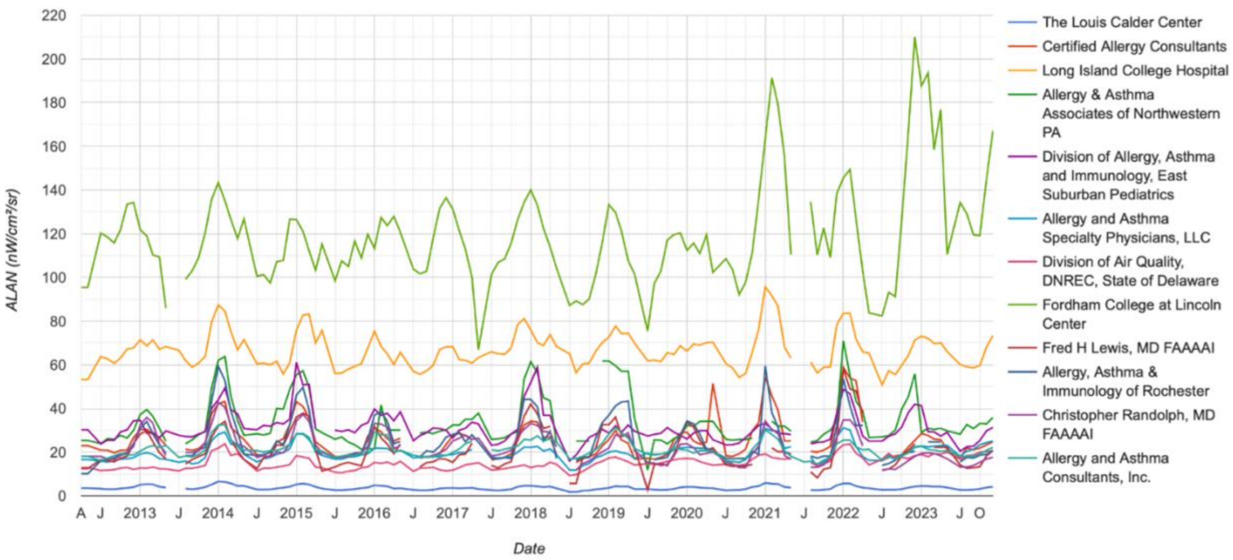

Fig. S3. Monthly ALAN levels at pollen stations during 2012-2023. ALAN is measured in $nW/cm^2/sr$ from monthly average radiance composite images using nighttime data from the Visible Infrared Imaging Radiometer Suite (VIIRS) Day/Night Band (DNB), and the 3-month smoothed ALAN is shown here.

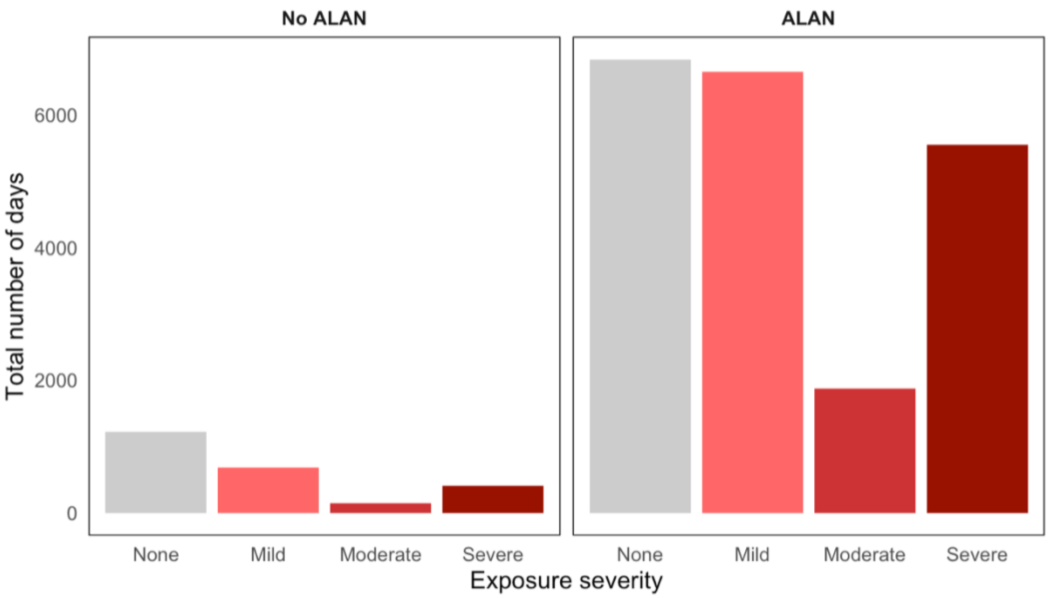

Fig. S4. Total number of days across exposure severity levels under no ALAN and ALAN conditions. "None" (<10 grains/m³), "Mild" (10–49 grains/m³), "Moderate" (50–89 grains/m³), and "Severe" (≥90 grains/m³). These severity levels were aggregated across all pollen seasons for sites categorized as "No ALAN" (<10 nW/cm²/sr) or "ALAN" (>10 nW/cm²/sr).

Table S1. Results of simple linear regression models assessing the relationship between mean annual artificial light at night (ALAN) and pollen season metrics. Pollen season metrics include start of pollen season (SPS), end of pollen season (EPS), and pollen season length (PSL). Each model includes ALAN as the sole predictor. Estimates, standard errors, t-values, and p-values are reported alongside model R^2, adjusted R^2, and residual standard error.

Response Variable	Predictor	Estimate	Std. Error	t-value	p-value	R^2	Adjusted R^2	Residual SE
SPS (DOY)	ALAN	–0.16634	0.055	–3.04	0.0031			
	Intercept	82.36	2.81	29.26	< 2e-16	0.090	0.080	17.14
EPS (DOY)	ALAN	0.17376	0.044	3.96	0.0001			
	Intercept	274.09	2.26	121.40	< 2e-16	0.144	0.135	13.75
PSL (Days)	ALAN	0.34009	0.081	4.21	5.99e-05			
	Intercept	191.73	4.16	46.14	< 2e-16	0.160	0.151	25.31

Table S2. Results of interaction term tests for pollen season metrics. Coefficients and p-values for two modeling approaches: Single-interaction models (models containing only one two-way interaction term at a time, with all main effects retained) and the full interaction model (model containing all two-way interactions among predictors simultaneously). The pollen metrics are start of pollen season (SPS), end of pollen season (EPS), and pollen season length (PSL). The predictors are artificial light at night (ALAN), temperature (Temp), and precipitation (Prcp).

Model Type	Metric	Term	Estimate	p-value
Single	SPS	ALAN x Temp	-0.013	0.794
	EPS	ALAN x Temp	0.064	0.180
	PSL	ALAN x Temp	0.077	0.341
	SPS	ALAN x Prcp	-0.002	0.702
	EPS	ALAN x Prcp	-0.001	0.819
	PSL	ALAN x Prcp	0.001	0.919
	SPS	Temp x Prcp	0.025	0.880
	EPS	Temp x Prcp	0.084	0.601
	PSL	Temp x Prcp	0.058	0.830
Full	SPS	ALAN x Temp	-0.0158	0.7567
		ALAN x Prcp	-0.0035	0.6163
		Temp x Prcp	0.0691	0.7120
	EPS	ALAN x Temp	0.0606	0.2110
		ALAN x Prcp	-0.0026	0.6930
		Temp x Prcp	0.0902	0.6100
	PSL	ALAN x Temp	0.0764	0.3540
		ALAN x Prcp	0.0009	0.9370
		Temp x Prcp	0.0211	0.9440

Table S3. Taxonomic groupings of pollen data used in the analysis. All pollen and spore taxa recorded in the NAB dataset were classified into groups of trees, grasses, and weeds, based on botanical characteristics and common allergenic profiles.

**Group**	**Taxa**
Trees	*Acer, Alnus, Betula, Carpinus / Ostrya, Carya, Celtis, Corylus, Cupressaceae, Fagus, Fraxinus, Juglans, Ligustrum, Liquidambar, Morus, Myrica, Olea, Other Tree Pollen, Pinaceae, Platanus, Populus, Prosopis, Pseudotsuga, Quercus, Salix, Tilia, Tsuga, Ulmus*
Grasses	*Gramineae / Poaceae, Other Grass Pollen (non-grass graminoids)*
Weeds	*Ambrosia, Arecaceae, Artemisia, Asteraceae (excluding Ambrosia and Artemisia), Chenopodiaceae/Amaranthaceae, Eupatorium, Plantago, Rumex, Typha, Urticaceae, Other Weed Pollen*

Table S4. Variance inflation factors for predictors used in three linear models (SPS, EPS, and PSL) assessing the influence of mean annual ALAN, mean annual temperature, and total annual precipitation. All variance inflation factor values were below the common threshold of 5, indicating low multicollinearity among predictors.

Model	Variable	VIF
Start of pollen season	ALAN	1.051
	Temperature	1.106
	Precipitation	1.063
End of pollen season	ALAN	1.051
	Temperature	1.106
	Precipitation	1.063
Pollen season length	ALAN	1.051
	Temperature	1.106
	Precipitation	1.063

Table S5. Assessment of linearity for ALAN effects: comparison of GAM smooths (k=5) vs. linear terms for start of pollen season, end of pollen season, and pollen season length. The table reports edf (smooth), AIC (spline), AIC (linear), and ΔAIC (spline - linear), as well as the decision in model selection.

Outcome	edf (smooth)	AIC (spline)	AIC (linear)	ΔAIC (spline - linear)	Decision
Start of pollen season	~1.0	778.533	778.467	0.066	Linear
End of pollen season	~1.0	749.552	749.547	0.005	Linear
Pollen season length	~1.0	855.880	855.873	0.006	Linear

Table S6. Influence and robustness checks for ALAN effects on start of pollen season, end of pollen season, and pollen season length. Models shown as OLS, OLS excluding high-influence observations (Cook's > 4/n), and Huber-robust.

**Outcome**	**n removed (Cook's > 4/n)**	**β ALAN (OLS)**	**β ALAN (no-influence)**	**β_ALAN (robust)**	**Decision**
Start of pollen season	6	0.014577	-0.151034	-0.082897	Inference unchanged; OLS
End of pollen season	4	0.343988	0.320815	0.284292	Inference unchanged; OLS
Pollen season length	7	0.329411	0.422024	0.454017	Inference unchanged; OLS

Table S7. Contemporaneous versus 1-year lagged ALAN in station-fixed models for start of pollen season, end of pollen season, and pollen season length. The table reports the ALAN coefficient for contemporaneous and lag-1 models, the ΔAIC (lag - now), and the decision rule (|ΔAIC| > 2). Station fixed effects and year dummies are included.

Outcome	β now (ALAN)	β lag (ALAN)	ΔAIC (lag - now)	Decision
Start of pollen season	-0.099	0.304	3.571	Prefer contemporaneous
End of pollen season	0.206	0.080	0.042	Similar
Pollen season length	0.305	-0.224	-0.978	Similar

Table S8. Station-level trends in ALAN levels at pollen stations (2012-2023). ALAN is 3-month smoothed value and measured in nW/cm^2/sr from monthly average radiance composite images using nighttime data from the Visible Infrared Imaging Radiometer Suite (VIIRS) Day/Night Band (DNB). Slopes are derived from linear regression of ALAN vs. years. The p-values are from two-sided tests of the null hypothesis of zero slope.

Pollen station	Slope (nW/cm^2/sr/ year)	p-value
The Louis Calder Center	-0.032	0.203
Certified Allergy Consultants	0.149	0.521
Long Island College Hospital	0.122	0.578
Allergy & Asthma Associates of Northwestern PA	0.115	0.703
Division of Allergy, Asthma, and Immunology, East Suburban Pediatrics	-0.376	0.050
Allergy and Asthma Specialty Physicians, LLC	0.274	<0.01
Division of Air Quality, DNREC, State of Delaware	0.477	<0.001
Fordham College at Lincoln Center	2.133	<0.001
Fred H Lewis, MD FAAAAI	0.024	0.925
Allergy, Asthma, & Immunology of Rochester	-0.217	0.437
Christopher Randolph, MD FAAAAI	-0.646	<0.001
Allergy and Asthma Consultants, Inc.	-0.121	0.227

Table S9. Summary of taxonomic composition similarity between pollen stations based on Bray-Curtis dissimilarity of Hellinger-transformed data. Average dissimilarity and similarity values are shown, alongside a qualitative interpretation of compositional differences.

Avg. Dissimilarity	Avg. Similarity	Conclusion
0.382	0.618	Moderately similar
